# Can a score derived from the Critical Care Minimum Data Set be used as a marker of organ dysfunction? – a pilot study

**DOI:** 10.1186/1756-0500-2-77

**Published:** 2009-05-06

**Authors:** Tim W Felton, Rebecca Sander, Mo Al-Aloul, Paul Dark, Andrew M Bentley

**Affiliations:** 1 University Department of Intensive Care Medicine, Salford Royal NHS Foundation Trust, Greater Manchester, M6 8HD, UK; 2 North West Lung Centre, Wythenshawe Hospital, University of South Manchester NHS Trust, Manchester, UK

## Abstract

**Background:**

The aim of this study was to develop a simple organ score derived from the Critical Care Minimum Data Set (CCMDS) to compare with the Sequential Organ Failure Assessment (SOFA) score, a previously validated score of organ dysfunction.

**Findings:**

The CCMDS collects data regarding the support of seven organ systems. To create a CCMDS derived score each level of organ support was allocated a numerical value. SOFA scores were collected retrospectively from each patient in the study. Data was collected in 50 sequential admissions over the first 5 days of their admission. This generated a total of 147 pairs of data for comparison.

Scatter plots and Spearman's rank correlation coefficient suggest a weak positive association between our CCMDS-derived score and the SOFA score. Daily Bland-Altman plots reveal minimal bias between the score but wide limits of agreement.

**Conclusion:**

Our CCMDS-derived score cannot be regarded as an indicator of severity of organ dysfunction and cannot replace SOFA scores when a daily marker of organ dysfunction is required.

## Findings

The Critical Care Minimum Data Set (CCMDS) was introduced in April 2006 and collates data daily regarding all patients, excluding neonates, in level 2 or 3 critical care beds in England and Wales. The CCMDS was developed by the Critical Care Information Advisory Group and is endorsed by the Intensive Care Society. The data consists of 34 fields which allow calculation of admission duration, basic demographic information, level of care and category of intensive care unit (general, neurosurgical, cardiothoracic etc). Information is also collected regarding the number of organs supported and, in the case of the cardiovascular and respiratory systems, the level of support. Data is currently submitted to the Intensive Care National Audit and Research Centre (ICNARC) as part of the Case Mix Programme. During 2008 data collection became mandatory and will be used to calculate a payment tariff under the Payment by Results (PbR) scheme within all NHS hospitals.

Our aim was to develop a simple organ score derived from the CCMDS to compare with a previously validated score of organ dysfunction. The Sequential Organ Failure Assessment (SOFA) score is a very commonly used scoring system designed to follow morbidity of critically ill patients[1]. It records severity of dysfunction of six organ systems. Improvement in organ dysfunction scores, such as SOFA, monitor improvements in a patient's condition following an intervention or as an end point for clinical trials. More recently, organ dysfunction scores have been validated, not only as an initial indicator of outcome on admission to ICU, but also as a predictor of mortality, dependant on changes in daily scores during a patient's ICU admission [2-4]. Organ dysfunction scores are time consuming and expensive to collect, requiring additional clerical support so a CCMDS-derived score could represent a saving of both time and money to UK intensive care units especially those involved in clinical trials.

The CCMDS collects data regarding the support of seven organ systems. The support of the cardiovascular and respiratory systems is further divided into basic and advanced support, whilst the other five systems are either supported or not. Table 1 defines each method of organ support [5]. SOFA scores were collected retrospectively from each patient in the study. The study ICU routinely documents Bloomsbury sedation scores rather than Glasgow Coma Scores (GCS) as recorded in the SOFA score[6]. Table 2 illustrates the scheme used to convert the sedation score to the neurological component of the SOFA score. In order to create a CCMDS derived score each level of organ support was allocated a numerical value (Table 3) with basic support scoring 1 and advanced support scoring 2. We did not allocate a particular weighting to any of the scores.

**Table 1:** Summary of definitions of levels of organ support in the critical care minimum data set [5].

**Organ system**	**Definition of level of support**
**Basic respiratory**	• > 50% oxygen delivered by face mask
	• Close observation due to the potential for acute deterioration
	• Physiotherapy or suction to clear secretions at least two hourly
	• Patients recently extubated after a prolonged period of intubation and mechanical ventilation
	• Mask CPAP or non-invasive ventilation.
	• Patients who are intubated to protect the airway but needing no ventilatory support and who are otherwise stable

**Advanced respiratory**	• Invasive mechanical ventilatory support
	• Extracorporeal respiratory support

**Basic cardiovascular**	• Treatment of circulatory instability due to hypovolaemia
	• Use of a CVP line for basic monitoring or central access
	• Use of an arterial line for basic monitoring or sampling
	• Single intravenous vasoactive
	• Intravenous drugs to control cardiac arrhythmias
	• Non-invasive measurement of cardiac

**Advanced cardiovascular**	• Multiple intravenous vasoactive and/or rhythm controlling drugs
	• Patients resuscitated after cardiac arrest where intensive therapy is considered clinically appropriate.
	• Observation of cardiac output and derived
	• Intra aortic balloon pumping.
	• Temporary cardiac
	• Placement of a gastrointestinal tonometer

**Renal**	• Acute renal replacement therapy

**Neurological**	• Central nervous system depression sufficient to prejudice the airway and protective reflexes
	• Invasive neurological monitoring
	• Severely agitated or epileptic patients requiring constant nursing attention and/or heavy sedation

**Gastrointestinal**	• Feeding with parenteral or enteral nutrition

**Dermatological**	• Patients with major skin rashes, exfoliation or burns
	• Use of multiple, large trauma dressings
	• Use of complex dressings

**Liver**	• Extracorporeal liver replacement device

**Table 2:** System used for conversion from sedation score to neurological component of the SOFA score.

	**Sedation score**	**Neurological component** **of SOFA score**
**Agitated/restless**	3	1 (GCS 13–14)

**Awake/uncomfortable**	2	1 (GCS 13–14)

**Awake/calm**	1	0 (GCS 15)

**Roused by voice**	0	2 (GCS 10–12)

**Roused by movement**	-1	3 (GCS 6–9)

**Rouse by pain**	-2	3 (GCS 6–9)

**Unrousable**	-3	4 (GCS < 6)

**Paralysed**	P	4 (GCS < 6)

**Table 3:** The CCMDS-derived score

		**Score**
Respiratory support	No support	0

	Basic support	1

	Advance support	2

Cardiovascular support	No support	0

	Basic support	1

	Advance support	2

Renal support	No support	0

	Support	1

Neurological support	No support	0

	Support	1

GI support	No support	0

	Support	1

Dermatological support	No support	0

	Support	1

Liver support	No support	0

	Support	1

Our aim was to retrospectively collect SOFA scores and the CCMDS-derived score on fifty admissions to a teaching hospital general intensive care unit from January 1st 2007. The local ethics committee was informed and indicated that no formal approval of this retrospective study was required. Data was collected from day 0 to day 5 of each patient's admission. Seven of the original fifty patients had insufficient data to allow calculation of either a SOFA score or CCMDS derived score. In total 147 pairs of data, both SOFA and CCMDS-derived scores were collected with each patient contributing between two and six data points. Data was initially analysed for correlation and agreement by plotting a scatter plot of the scores of one method against those of the other for each day of admission. Subsequently a Spearman's rank test and a Bland and Altman plot were performed on data collected for each day to assess whether these two methods can be used interchangeably[7].

**Figure 1:**
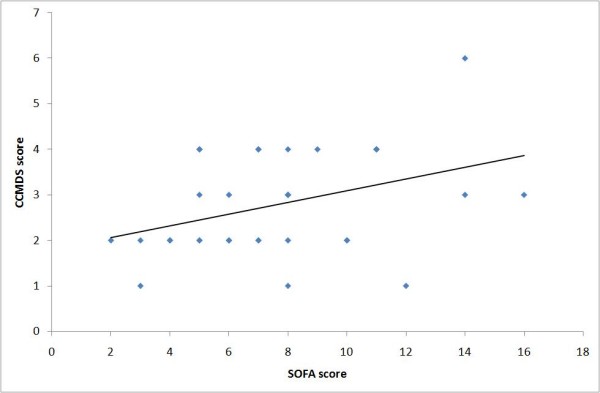
**Comparison of CCMDS-derived score and the SOFA score on day 0**. Figure 1 shows a XY scatter plot comparing the CCMDS derived score with the SOFA score on day 0.

**Figure 2:**
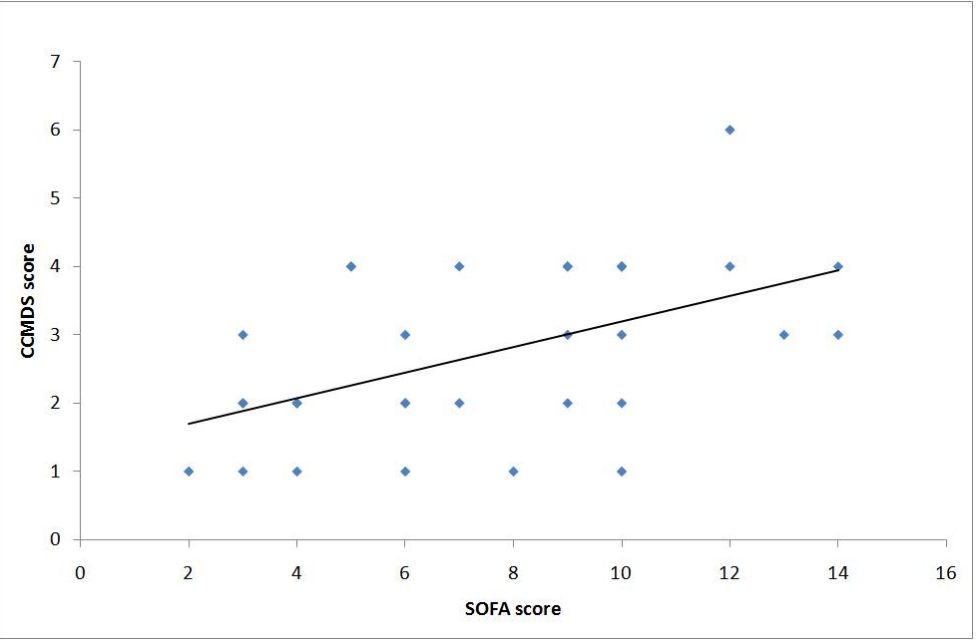
**Comparison of CCMDS-derived score and the SOFA score on day 1**. Figure 2 shows a XY scatter plot comparing the CCMDS derived score with the SOFA score on day 1.

Visual inspection of the graphs comparing the CCMDS-derived score and SOFA score suggests a weak positive association. The graphs for day 0 and day 1 are illustrated in figure 1 and 2. The weak positive correlation is confirmed using Spearman's rank test, applied to data collected on each day. This gives a correlation coefficient between r = 0.350 to r = 0.609 with the p value for each day (shown in table 4), suggesting a significant correlation (range of p values p = 0.002 to p = 0.047), with the exception of day 2 (p = 0.054). This would suggest that the ranking of patients from high to low risk is similar for both scores.

**Table 4:** Spearman's rank correlation coefficient for the CCMDS-derived score compared to SOFA scores for day 0 to day 5.

**Day**	**Correlation coefficient (r value)**	**r^2^**	**p value**
**0**	0.350	0.123	0.046

**1**	0.516	0.266	0.002

**2**	0.398	0.158	0.054

**3**	0.536	0.287	0.015

**4**	0.529	0.280	0.035

**5**	0.609	0.371	0.047

**Figure 3:**
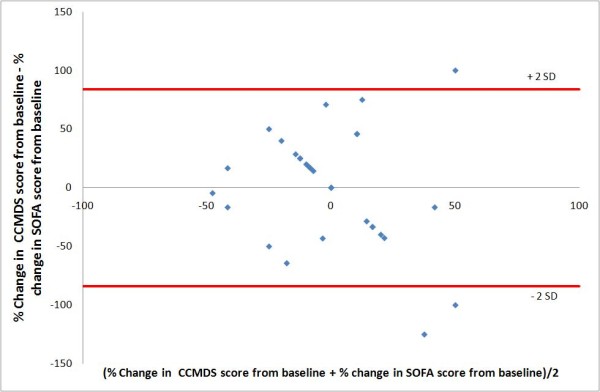
**Bland and Altman plot comparing the CCMDS-derived score and SOFA day 0 to day 1**. Figure 3 shows a Bland and Altman plot comparing the difference in percentage change, from day 0 to day 1, in the CCMDS-derived score and SOFA score with the average of the CCMDS-derived score and SOFA score. the limits of agreement are indicated by the red lines.

Levels of agreement were checked using a Bland and Altman plot for each day of data collected. As the total score for both score was not identical a percentage change in the score was used. An example of the Bland and Altman plot is shown in figure 3 comparing the changes in scores between day 0 and day 1. Both scores were expressed as a percentage change from the baseline admission score. There is a broad spread of results in the graphs produced for each day of data collected, reflecting a poor level of agreement. The average overall bias between the two scores, in the percentage change from baseline was minimal on day 1 to 0 i.e. 1%. However the 95% limits of agreement were -84% to 86%. This wide margin of disagreement could be expected as the SOFA score has a much greater range than the CCMDS-derived score. It is therefore likely to be a more sensitive measure of change. Similar results were obtained for data on all the other days. The limits of agreement for each day as a percentage change from baseline are shown in table 5.

**Table 5:** Levels of agreement derived from the Bland and Altman plots for the 5 days of admission.

	**Bias**	**Limits of Agreement**
**Day 0 to 1**	1%	-84% to 86%

**Day 0 to 2**	4%	-58% to 65%

**Day 0 to 3**	7%	-49% to 63%

**Day 0 to 4**	9%	-59% to 78%

**Day 0 to 5**	11%	-50% to 72%

The aim of our CCMDS-derived score was to produce a simple, daily scoring system that would monitor severity of organ dysfunction but require no additional administrative input and therefore no additional cost implications. The results show a statistical correlation between the CCMDS-derived score and SOFA score as indicators of organ dysfunction, confirming the ranking of patients is similar for the two scoring systems. The r^2 ^values suggest that factors not included in the SOFA score affect our CCMDS-derived score. However, the Bland and Altman plots display a poor degree of agreement between the two scores but with minimal overall bias.

Based on this pilot data from 43 patients our CCMDS-derived score cannot be regarded as an indicator of the severity of organ dysfunction when compared with the SOFA score. It is our view, at present in critically ill patients, an organ dysfunction score derived form the CCMDS cannot replace the SOFA score in studies where a daily indicator of the severity of organ dysfunction is required. This may be explained by the different purpose of each score. The SOFA score was developed and validated as a tool to quantify organ dysfunction. In contrast, the CCMDS was developed to calculate the level of dependency of a patient as a way of calculating remuneration for critical care units. Whilst the level of organ dysfunction is related to the dependency of the patient the two scores quantify two distinct entities.

The sample size was set at 50 as this was a pilot study to determine the feasibility of using a CCMDS-derived score as an organ dysfunction score. Although a larger sample size may reduce the chance of a negative result being a type II error we feel that this is unlikely in view of the consistency of the results. The small sample size and original premise of this pilot study makes more detailed statistical analysis beyond the remit of this study. This does, however, limit the conclusions that can be drawn from the data. Based on the results of this study it is unlikely that a large prospective study with detailed statistical analysis would produce a satisfactory new organ dysfunction score based on the CCMDS. Specific weaknesses of the study are the derived neurological component of the SOFA score, its retrospective nature and small sample size. The Glasgow Coma Score is not routinely collected in the study intensive care unit thereby necessitating a conversion of sedation scores to GCS. A prospective study would be able to record GCS. However this would defeat the main intention of the study to record a score that requires no additional administrative costs. This study is to our knowledge, the first attempt to use the critical care minimum data set to predict organ dysfunction.

## Abbreviations

CCMDS: Critical Care Minimum Data Set; SOFA: Sequential Organ Failure Assessment; ICNARC: Intensive Care National Audit and Research Centre; PbR: Payment by Results; GCS: Glasgow Coma Scale.

## Competing interests

The authors declare that they have no competing interests.

## Authors' contributions

TWF participated in the study design, data collection, statistical analysis and drafted the manuscript. RS participated in the statistical analysis and helped draft the manuscript. MA participated in the study design, coordination and data collection. PMD participated in the study design, statistical analysis and help draft the manuscript. AB participated in the study design, statistical analysis and helped draft the manuscript. All authors read and approved the final manuscript.
